# Lowering of Circulating Sclerostin May Increase Risk of Atherosclerosis and Its Risk Factors: Evidence From a Genome‐Wide Association Meta‐Analysis Followed by Mendelian Randomization

**DOI:** 10.1002/art.42538

**Published:** 2023-08-03

**Authors:** Jie Zheng, Eleanor Wheeler, Maik Pietzner, Till F. M. Andlauer, Michelle S. Yau, April E. Hartley, Ben Michael Brumpton, Humaira Rasheed, John P. Kemp, Monika Frysz, Jamie Robinson, Sjur Reppe, Vid Prijatelj, Kaare M. Gautvik, Louise Falk, Winfried Maerz, Ingrid Gergei, Patricia A. Peyser, Maryam Kavousi, Paul S. de Vries, Clint L. Miller, Maxime Bos, Sander W. van der Laan, Rajeev Malhotra, Markus Herrmann, Hubert Scharnagl, Marcus Kleber, George Dedoussis, Eleftheria Zeggini, Maria Nethander, Claes Ohlsson, Mattias Lorentzon, Nick Wareham, Claudia Langenberg, Michael V. Holmes, George Davey Smith, Jonathan H. Tobias

**Affiliations:** ^1^ Department of Endocrine and Metabolic Diseases, Shanghai Institute of Endocrine and Metabolic Diseases, Ruijin Hospital, Shanghai Jiao Tong University School of Medicine, Shanghai, China, and Shanghai National Clinical Research Center for Metabolic Diseases, Key Laboratory for Endocrine and Metabolic Diseases of the National Health Commission of the People's Republic of China, Shanghai Key Laboratory for Endocrine Tumor, State Key Laboratory of Medical Genomics, Ruijin Hospital, Shanghai Jiao Tong University School of Medicine, Shanghai, China, and MRC Integrative Epidemiology Unit (IEU), Bristol Medical School, University of Bristol Bristol UK; ^2^ MRC Epidemiology Unit, Institute of Metabolic Science University of Cambridge School of Clinical Medicine Cambridge UK; ^3^ MRC Epidemiology Unit, Institute of Metabolic Science, University of Cambridge School of Clinical Medicine, Cambridge, UK, and Computational Medicine, Berlin Institute of Health at Charité–Universitätsmedizin Berlin Berlin Germany; ^4^ Department of Neurology, Klinikum rechts der Isar, School of Medicine Technical University of Munich Munich Germany; ^5^ Marcus Institute for Aging Research, Hebrew SeniorLife Harvard Medical School Boston Massachusetts; ^6^ MRC IEU, Bristol Medical School University of Bristol Bristol UK; ^7^ K.G. Jebsen Center for Genetic Epidemiology, Department of Public Health and Nursing, NTNU, Norwegian University of Science and Technology, Trondheim, and HUNT Research Centre, Department of Public Health and Nursing, NTNU Norwegian University of Science and Technology Levanger Norway; ^8^ MRC IEU, Bristol Medical School, University of Bristol, Bristol, UK, and HUNT Research Centre, Department of Public Health and Nursing, NTNU, Norwegian University of Science and Technology, Levanger, Norway, and Division of Medicine and Laboratory Sciences, Faculty of Medicine University of Oslo Oslo Norway; ^9^ MRC IEU, Bristol Medical School, University of Bristol, Bristol, UK, and Institute for Molecular Bioscience, The University of Queensland, Brisbane, Queensland, Australia, and The University of Queensland Diamantina Institute The University of Queensland Brisbane Queensland Australia; ^10^ MRC IEU, Bristol Medical School, University of Bristol, and Musculoskeletal Research Unit University of Bristol Bristol UK; ^11^ Unger‐Vetlesen Institute, Lovisenberg Diaconal Hospital and Department of Plastic and Reconstructive Surgery, Oslo University Hospital and Department of Medical Biochemistry Oslo University Hospital Oslo Norway; ^12^ Department of Internal Medicine Erasmus MC University Medical Center Rotterdam The Netherlands; ^13^ Unger‐Vetlesen Institute Lovisenberg Diaconal Hospital Oslo Norway; ^14^ Clinical Institute of Medical and Chemical Laboratory Diagnostics, Medical University of Graz, Austria, and SYNLAB Academy, SYNLAB Holding Deutschland GmbH and Vth Department of Medicine (Nephrology, Hypertensiology, Rheumatology, Endocrinology, Diabetology), Medical Faculty Mannheim University of Heidelberg Mannheim Germany; ^15^ Vth Department of Medicine (Nephrology, Hypertensiology, Rheumatology, Endocrinology, Diabetology), Medical Faculty Mannheim, University of Heidelberg, Mannheim, and Therapeutic Area Cardiovascular Medicine Boehringer Ingelheim International GmbH Ingelheim Germany; ^16^ Department of Epidemiology, School of Public Health University of Michigan Ann Arbor; ^17^ Department of Epidemiology, Erasmus MC University Medical Center Rotterdam The Netherlands; ^18^ Human Genetics Center, Department of Epidemiology, Human Genetics, and Environmental Sciences, School of Public Health The University of Texas Health Science Center at Houston; ^19^ Center for Public Health Genomics, Department of Public Health Sciences University of Virginia Charlottesville; ^20^ Central Diagnostics Laboratory, Division of Laboratories, Pharmacy, and Biomedical Genetics, University Medical Center Utrecht Utrecht University Utrecht the Netherlands; ^21^ Cardiology Division, Department of Medicine Massachusetts General Hospital Boston; ^22^ Clinical Institute of Medical and Chemical Laboratory Diagnostics Medical University of Graz Graz Austria; ^23^ SYNLAB Academy, SYNLAB Holding Deutschland GmbH Mannheim Germany; ^24^ Department of Nutrition and Dietetics, School of Health Science and Education Harokopio University Athens Greece; ^25^ Institute of Translational Genomics, Helmholtz Zentrum München, German Research Center for Environmental Health, Neuherberg, and Technical University of Munich (TUM) and Klinikum Rechts der Isar TUM School of Medicine Munich Germany; ^26^ Sahlgrenska Osteoporosis Centre, Centre for Bone and Arthritis Research, Department of Internal Medicine and Clinical Nutrition, Institute of Medicine, University of Gothenburg and Bioinformatics and Data Centre, Sahlgrenska Academy University of Gothenburg Gothenburg Sweden; ^27^ Sahlgrenska Osteoporosis Centre, Centre for Bone and Arthritis Research, Department of Internal Medicine and Clinical Nutrition, Institute of Medicine University of Gothenburg Gothenburg Sweden; ^28^ Sahlgrenska Osteoporosis Centre, Institute of Medicine, Sahlgrenska Academy, University of Gothenburg, Gothenburg, Sweden, and Region Västra Götaland, Geriatric Medicine, Sahlgrenska University Hospital, Mölndal, Sweden, and Mary McKillop Institute for Health Research Australian Catholic University Melbourne Victoria Australia; ^29^ MRC IEU, Bristol Medical School, University of Bristol, and Medical Research Council Population Health Research Unit, University of Oxford, and Clinical Trial Service Unit & Epidemiological Studies Unit, Nuffield Department of Population Health University of Oxford, and National Institute for Health Research, Oxford Biomedical Research Centre, Oxford University Hospital Oxford UK

## Abstract

**Objective:**

In this study, we aimed to establish the causal effects of lowering sclerostin, target of the antiosteoporosis drug romosozumab, on atherosclerosis and its risk factors.

**Methods:**

A genome‐wide association study meta‐analysis was performed of circulating sclerostin levels in 33,961 European individuals. Mendelian randomization (MR) was used to predict the causal effects of sclerostin lowering on 15 atherosclerosis‐related diseases and risk factors.

**Results:**

We found that 18 conditionally independent variants were associated with circulating sclerostin. Of these, 1 *cis* signal in *SOST* and 3 *trans* signals in *B4GALNT3*, *RIN3*, and *SERPINA1* regions showed directionally opposite signals for sclerostin levels and estimated bone mineral density. Variants with these 4 regions were selected as genetic instruments. MR using 5 correlated *cis*‐SNPs suggested that lower sclerostin increased the risk of type 2 diabetes mellitus (DM) (odds ratio [OR] 1.32 [95% confidence interval (95% CI) 1.03–1.69]) and myocardial infarction (MI) (OR 1.35 [95% CI 1.01–1.79]); sclerostin lowering was also suggested to increase the extent of coronary artery calcification (CAC) (β = 0.24 [95% CI 0.02–0.45]). MR using both *cis* and *trans* instruments suggested that lower sclerostin increased hypertension risk (OR 1.09 [95% CI 1.04–1.15]), but otherwise had attenuated effects.

**Conclusion:**

This study provides genetic evidence to suggest that lower levels of sclerostin may increase the risk of hypertension, type 2 DM, MI, and the extent of CAC. Taken together, these findings underscore the requirement for strategies to mitigate potential adverse effects of romosozumab treatment on atherosclerosis and its related risk factors.

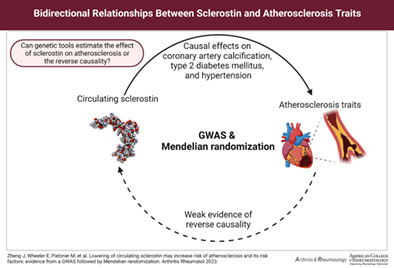

## INTRODUCTION

The inhibition of sclerostin is a therapeutic approach to increasing bone mineral density (BMD) and lowering fracture risk in patients with osteoporosis. However, 2 phase III trials of romosozumab, a first‐in‐class monoclonal antibody that inhibits sclerostin, reported higher numbers of cardiovascular serious adverse events in the romosozumab‐treated group as compared to the comparator (1, 2). However, a similar imbalance of cardiovascular disease (CVD) was not seen in another study comparing romosozumab to placebo (3). Possibly, these different results reflect a beneficial effect of bisphosphonate treatment on risk of CVD. For example, zoledronate, a bisphosphonate, has been found to decrease all‐cause mortality, to which reduced CVD mortality may contribute (4). However, a beneficial effect on mortality was not borne out in a meta‐analysis of drug trials of zoledronate and other bisphosphonates (5). The role of sclerostin in the vasculature is unknown, though some studies have shown that its inhibition may promote vascular calcification, which could increase the risk of CVD (6). Given these concerns regarding CVD safety, marketing authorization for romosozumab indicates previous myocardial infarction (MI) or stroke as contraindications, underlying the urgent need to understand the causal role of sclerostin lowering on CVD outcomes, thereby providing physicians and patients with more credible information when balancing the risks and benefits of treatment.

Mendelian randomization (MR) uses genetic variants as proxies for an exposure to estimate the causal effect of a modifiable risk factor on a disease (7), which minimizes the bias from confounders or reverse causality. In a recent MR study using BMD‐associated variants in the *SOST* region as a proxy for lower sclerostin levels, Bovijn et al found genetic evidence consistent with a potential adverse effect of sclerostin lowering on CVD‐related events (8). However, some weaknesses of this study were discussed. For example, the *SOST* single‐nucleotide polymorphisms (SNPs) used in this analysis are >30 kb downstream of the target gene. Another MR study using sclerostin gene expression in arterial and heart tissue as the exposure suggested little evidence of a causal effect of sclerostin expression on risk of MI or stroke (9).

An alternative approach to instrument selection is to use SNPs identified from a well‐powered genome‐wide association study (GWAS) of circulating sclerostin. In an earlier GWAS of sclerostin levels, we identified 3 *trans*‐acting genetic variants associated with sclerostin, including a top variant in the *B4GALNT3* region. However, we only observed marginal genetic associations in the *cis*‐*SOST* region and had limited power to examine causal relationships with extraskeletal phenotypes (10). Therefore, a more powerful GWAS of circulating sclerostin is needed to identify stronger genetic predictors, including those in the *cis*‐acting region. A further consideration is that a bidirectional causal pathway appears to exist between sclerostin and BMD, whereby reduced sclerostin levels cause an increase in BMD, whereas higher BMD increases sclerostin levels, possibly reflecting a feedback pathway (10). Therefore, findings from a sclerostin GWAS are potentially subject to misspecification of the primary phenotype (11), with genetic signals being detected which are primarily related to BMD rather than sclerostin. In order to mitigate against this, we aimed to implement a SNP selection strategy intended to identify SNPs with directionally opposite associations with sclerostin levels and BMD.

The goal of the present study was to examine potential safety concerns of sclerostin lowering on atherosclerosis and its risk factors using an MR approach, based on a set of instruments derived from an updated GWAS meta‐analysis of circulating sclerostin. To enable sufficient power to examine causal effects on extraskeletal phenotypes, we aimed to identify genetic predictors of sclerostin with good instrument strength, incorporating both *cis*‐ and *trans*‐acting variants, having assembled a sample over 3 times the size of our previous GWAS study (10).

## PATIENTS AND METHODS

### Summary of study design

**Figure 1:**
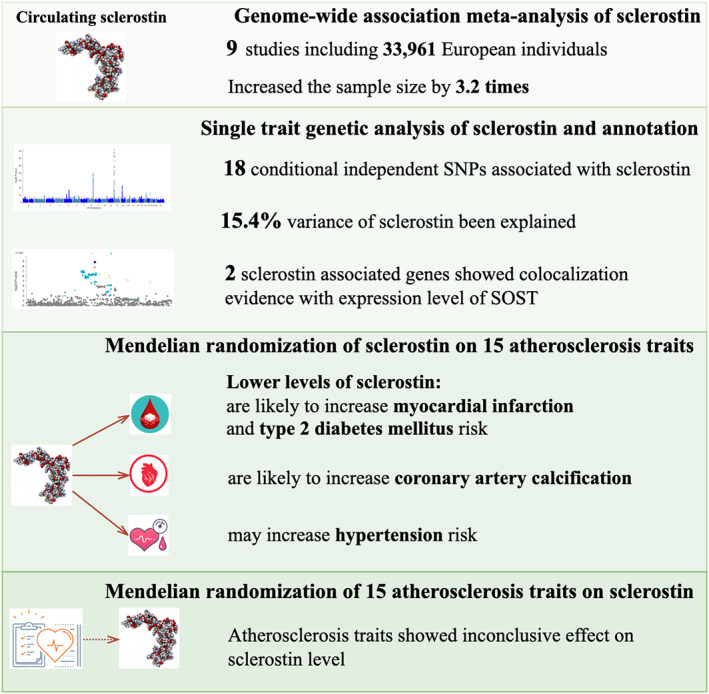
Summary of the design and results of the current study. This study included 4 major components: 1) meta‐analysis of genome‐wide association study of circulating sclerostin; 2) single trait genetic analysis and functional annotation of the top sclerostin signals; 3) Mendelian randomization and genetic correlation analysis of sclerostin on 15 atherosclerosis‐related diseases and risk factors traits; 4) bidirectional Mendelian randomization analysis of 15 atherosclerosis‐related diseases and risk factors on sclerostin. SNPs = single‐nucleotide polymorphisms; HDL‐C = high‐density lipoprotein cholesterol.

Figure 1 illustrates the design and participants of this study. First, we conducted a GWAS meta‐analysis and post‐GWAS follow‐up analyses of circulating sclerostin in 33,961 European individuals from 9 cohorts (12, 13, 14, 15, 16, 17, 18, 19, 20) (details of the cohorts and the characteristics of QC, imputation, and GWAS analysis of each cohort are presented in Supplementary Note 1, available on the *Arthritis & Rheumatology* website at http://onlinelibrary.wiley.com/doi/10.1002/art.42538). Second, we conducted MR analyses of circulating sclerostin using genetic instruments from both *cis* and *trans* regions (Supplementary Table 1, http://onlinelibrary.wiley.com/doi/10.1002/art.42538) and from the *cis* region only (Supplementary Table 2, http://onlinelibrary.wiley.com/doi/10.1002/art.42538). The outcomes are 15 atherosclerosis‐related diseases and risk factors (Supplementary Table 3, http://onlinelibrary.wiley.com/doi/10.1002/art.42538). Bidirectional MR was conducted for the 15 atherosclerosis‐related diseases and risk factors using genetic instruments shown in Supplementary Table 4 (http://onlinelibrary.wiley.com/doi/10.1002/art.42538).

### GWAS meta‐analysis of sclerostin

Sclerostin measures in the 9 cohorts were standardized to SD units. Each cohort ran a GWAS across all imputed or sequenced variants. Age and sex and the first 10 principal components were included as covariates in all models (except INTERVANL and LURIC). Details of the GWAS model, imputation panel, and covariates of each cohort are provided in Supplementary Note 1 (http://onlinelibrary.wiley.com/doi/10.1002/art.42538). We standardized the genomic coordinates to be reported on the NCBI build 37 (hg19) and alleles on the forward strand. Summary level quality control was conducted for each cohort separately using EasyQC; only individuals with European ancestry and genetic variants with minor allele frequency (MAF) >1% were selected for the meta‐analysis. Meta‐analysis (using a fixed‐effect model implemented in METAL [21]) was restricted to variants with a minimal sample size >10,000 individuals, MAF >1%, and high imputation quality score (R^2^ > 0.8 for variants imputed in MaCH [22] and INFO >0.8 for variants imputed in IMPUTE [23] [n = 11,680,861 variants]). Meta‐analysed *P* values lower than 5 × 10^−8^ were used as a heuristic to define “genome‐wide significant” associations. A random effects model meta‐analysis was also conducted using GWAMA version 2.2.2 (24). Heterogeneity was assessed using the I^2^ statistic and Cochran's Q test. The genetic effect estimate of SNP is presented in terms of the SD unit change in sclerostin levels, scaled from the difference in sclerostin level per effect allele.

#### Conditional analysis and genetic fine mapping

We carried out an approximate conditional and joint genome‐wide association analysis (GCTA‐COJO) to detect multiple independent association signals at each of the sclerostin loci (25). SNPs with high collinearity (correlation r^2^ > 0.9) were ignored, and those situated more than 10 Mb away were assumed to be in complete linkage equilibrium, which was the default setting for GCTA‐COJO (25). A reference sample of 8,890 unrelated individuals of Avon Longitudinal Study of Parents and Children (ALSPAC) mothers was used to model patterns of linkage disequilibrium between variants. Conditionally independent variants with *P* < 5 × 10^−8^ were annotated to the physically closest gene list in dbSNP (https://www.ncbi.nlm.nih.gov/SNP/).

### Functional mapping and annotation of sclerostin genetic association signals

#### Genetic colocalization of gene expression quantitative trait loci (eQTLs) and the sclerostin signals

We investigated whether the SNPs influencing serum sclerostin level were driven by *cis*‐acting effects on transcription by evaluating the overlap between the sclerostin‐associated SNPs and eQTLs within 500 kb of the gene identified using data derived from all tissue types from GTEx version 8 (26). Where eQTLs overlapped with sclerostin‐associated SNPs, we used genetic colocalization analysis (27) to estimate the posterior probability of each genomic locus containing a single variant affecting both circulating sclerostin and gene expression levels in different tissues.

We used Functional Mapping and Annotation of Genome‐Wide Association Studies (FUMA) (28), an integrative web‐based platform (http://fuma.ctglab.nl) containing information from 18 biological data repositories and tools, to characterize the genetic association signals of sclerostin, as well as gene set enrichment using the STARNET web app (29) (more details in Supplementary Note 2, http://onlinelibrary.wiley.com/doi/10.1002/art.42538).

### LD score regression analyses

#### Estimation of SNP heritability and genetic correlation using LD score regression

To estimate the amount of genomic inflation in the data due to residual population stratification, cryptic relatedness, and other latent sources of bias, we used LD score regression (30). We further quantified the overall SNP‐based heritability with LD score regression using a subset of 1.2 million HapMap SNPs (SNPs in the major histocompatibility complex region were removed due to complex LD structure). To estimate the genetic correlation between reduced sclerostin level and 15 atherosclerosis‐related diseases and risk factors and 2 bone phenotypes, we used a platform based on LD score regression as implemented in the online web utility LD Hub (31). The heritability estimate for small vessel disease was out‐of‐bounds (h^2^ < 0 due to limited sample size) and therefore small vessel disease was not included in the genetic correlation analysis.

### MR

#### Selection of genetic predictors for sclerostin

From the 18 conditionally independent sclerostin variants identified (Supplementary Table 1A, http://onlinelibrary.wiley.com/doi/10.1002/art.42538), we selected valid genetic predictors of sclerostin for the MR using 2 further criteria: (i) we only selected those genetic variants which showed single SNP MR evidence of sclerostin on BMD estimated using ultrasound in heel (eBMD; data from UK Biobank) (single SNP MR *P* value of sclerostin on eBMD less than the Bonferroni corrected *P* value cutoff [0.05/18 = 0.003]; Supplementary Table 1B, http://onlinelibrary.wiley.com/doi/10.1002/art.42538); (ii) the sclerostin‐reducing alleles of the genetic variants were associated with increased BMD level (i.e., these variants showed a negative Wald ratio for sclerostin on BMD). The final set of 4 genetic variants after applying these 2 additional criteria are listed in Supplementary Table 1C (http://onlinelibrary.wiley.com/doi/10.1002/art.42538). The analysis using these 4 variants is noted as the *cis* and *trans* analysis.

Due to the relevance of the *cis*‐acting variants, we conducted a sensitivity analysis using genetic variants restricted to *cis*‐acting variants (defined as ± 500 kb genomic region from the leading *SOST* SNP) (noted as the *cis*‐only analysis). Of the 41 SNPs associated with circulating sclerostin (at a regional‐wide association threshold < 1 × 10^−6^) in the *SOST* region (± 500 kb genomic region from rs66838809), LD clumping identified 5 correlated SNPs with LD r^2^ < 0.8 (Supplementary Table 2, http://onlinelibrary.wiley.com/doi/10.1002/art.42538). Such an LD r^2^ threshold was used here to avoid multicollinearity caused by SNPs in very high LD. The same instrument selection criteria of the *cis*+*trans* instruments were used here, where all 5 correlated variants showed robust and negative MR effects on eBMD (Supplementary Table 2, http://onlinelibrary.wiley.com/doi/10.1002/art.42538). Therefore, these correlated instruments were used in a generalized inverse variance weighted (IVW) approach that considered LD among instruments in the MR model (more details in a later section).

#### Outcome selection

We selected 8 atherosclerosis‐related diseases and 7 atherosclerosis‐related risk factors as primary outcomes. This list comprised 2 endpoints related to ischaemic heart disease (coronary artery disease [CAD] [32] and MI [33]), 4 stroke endpoints (ischemic stroke, cardioembolic stroke, large vessel disease, small vessel disease [34]), 2 measures of arterial calcification (coronary artery calcification [CAC] [35], abdominal aortic calcification [AAC] [36]), hypertension, type 2 DM (37), and 5 lipid/lipoprotein risk factors (low‐density lipoprotein [LDL] cholesterol, high‐density lipoprotein [HDL] cholesterol, triglycerides, apolipoprotein A‐I [Apo A‐I], and Apo B) (38). As reported in the original paper, MI was defined as International Classification of Diseases version‐10 (ICD‐10) codes I21, I22, I23, and I25.2, which included MI, and complications following acute MI. Doctor‐diagnosed and self‐reported MI were also included in the definition of MI (33). The definition for CAD was based on the following ICD‐10 codes: I21–I25 covering ischemic heart diseases and the following Office of Population Censuses and Surveys Classification of Interventions and Procedures, version 4 (OPCS‐4) codes: K40–K46, K49, K50, and K75, which includes replacement, transluminal balloon angioplasty, and other therapeutic transluminal operations on coronary artery and percutaneous transluminal balloon angioplasty and insertion of stent into coronary artery. Self‐reported CAD was also used in the definition (heart attack/MI, coronary angioplasty +/– stent, coronary artery bypass graft, and triple heart bypass). More information, including sample size, Mesh term, and consortium name for the outcomes are listed in Supplementary Table 3 (http://onlinelibrary.wiley.com/doi/10.1002/art.42538).

#### MR of sclerostin on atherosclerosis‐related phenotypes

For the *cis* and *trans* analysis, 4 selected variants robustly associated with circulating sclerostin within the *SOST*, *B4GALNT3*, *RIN3*, and *SERPINA1* regions were used as instruments. We applied a set of 2‐sample MR approaches (IVW, MR‐Egger, weighted median, single mode estimator, and weighted mode estimator) (39) to estimate the effect of circulating sclerostin on the 15 atherosclerosis‐related diseases and risk factors. Although we had a small number of relevant variants available for this analysis, we still used the MR‐Egger intercept term as an indicator of potential directional pleiotropy. Heterogeneity analysis of the instruments was conducted using Cochran's Q test.

For the *cis*‐only analysis, 5 correlated variants in the *cis* SOST region were selected as instruments. We applied a generalized IVW MR model followed by generalized Egger regression to account for LD structure between correlated SNPs in the *SOST* region and to boost statistical power (40). The generalized Egger regression intercept term was used as an indicator of potential directional pleiotropy.

#### Bidirectional MR analysis of atherosclerosis‐related phenotypes on sclerostin

To investigate the possibility of reverse causality between atherosclerosis‐related diseases and risk factors and circulating sclerostin level, we used genetic variants associated with 15 atherosclerosis‐related diseases and risk factors as genetic predictors (small vessel disease data has no valid genetic predictors, therefore, we were not able to perform bidirectional MR for this trait; for other genetic predictors, the genetic association data were extracted from relevant GWAS listed in Supplementary Table 4A, http://onlinelibrary.wiley.com/doi/10.1002/art.42538). We applied IVW, MR‐Egger, weighted median, single mode estimator, and weighted mode estimator (39). In addition, due to correlation between lipids and lipoproteins, we further applied a multivariable MR model (41) to estimate the independent effect of each lipid and lipoprotein on sclerostin (instruments listed in Supplementary Tables 4B and C, http://onlinelibrary.wiley.com/doi/10.1002/art.42538). To further validate the directionality of the analysis, we conducted Steiger filtering analysis of the 4 selected sclerostin instruments on the 15 atherosclerosis‐related diseases and risk factors.

All MR analyses were conducted using the MendelianRandomization R package and TwoSampleMR R package (github.com/MRCIEU/TwoSampleMR v0.5.6). The strength of the genetic predictors of sclerostin and the 15 atherosclerosis‐related diseases and risk factors were estimated using F statistics.

## RESULTS

### Genome‐wide association signals of circulating sclerostin

GWAS results of circulating sclerostin were available in 33,961 participants of European ancestry from a meta‐analysis of 9 cohorts (Supplementary Note 1, http://onlinelibrary.wiley.com/doi/10.1002/art.42538). Supplementary Figures 1 and 2 show the Manhattan and Q–Q plots of association results from the fixed‐effects meta‐analysis of sclerostin, respectively (http://onlinelibrary.wiley.com/doi/10.1002/art.42538). Little evidence of inflation was found in the test statistics (genomic inflation factor λ 1.082; LD score regression intercept = 1.023). Therefore, no genomic control correction was applied to the meta‐analysis results. Single‐trait LD score regression results showed that common variants included in the GWAS meta‐analysis explained 15.4% of the phenotypic variance of circulating sclerostin (SNP‐based heritability *h* ^*2*^ = 0.154, *P* = 3.01 × 10^−13^; all valid variants across the genome were used to estimate the heritability).

**Figure 2:**
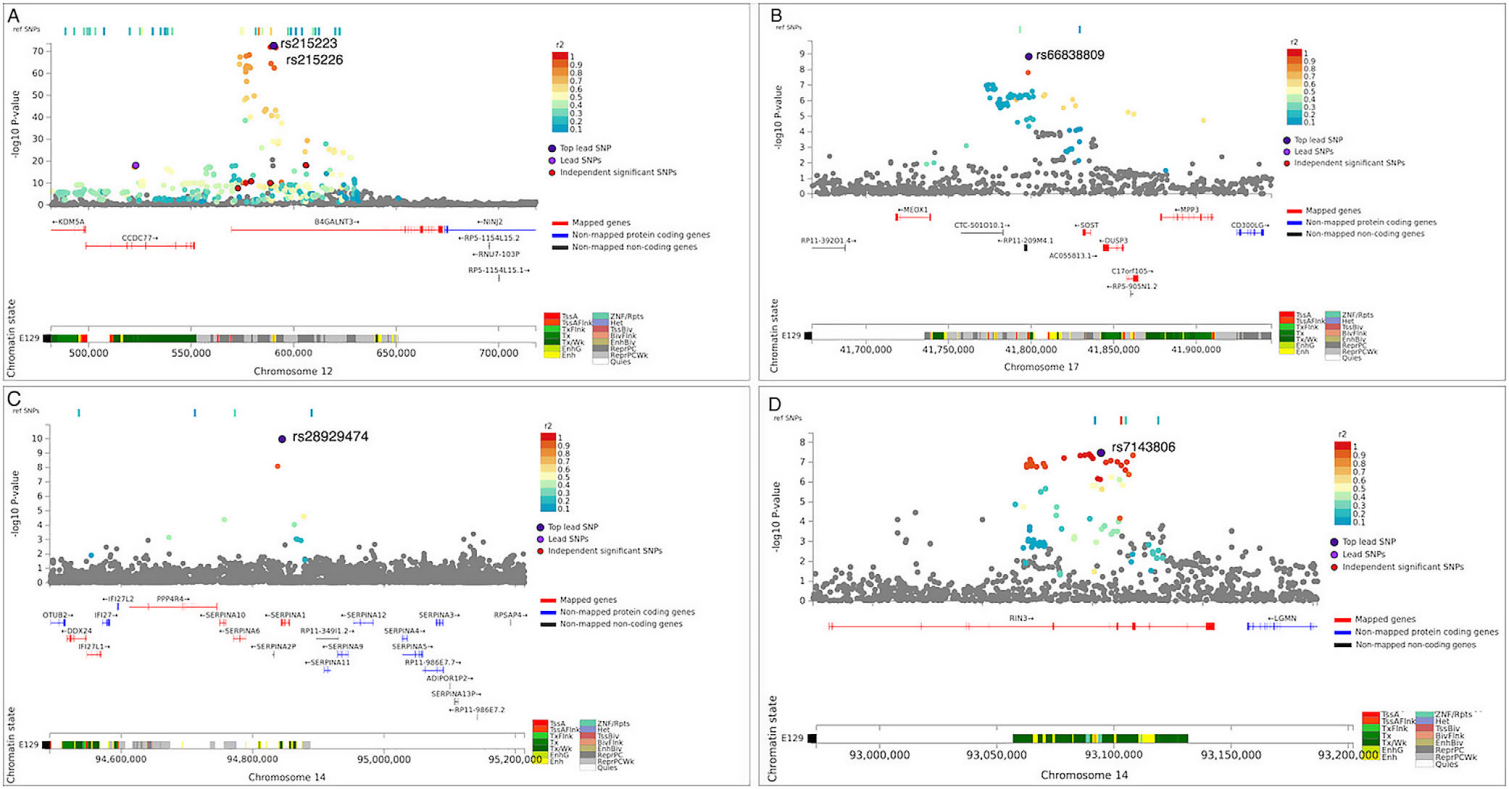
Genome‐wide association signals of circulating sclerostin. Regional plots for the *B4GLANT3* (**A**), *SOST* (**B**), *SERPINA1* (**C**), and *RIN3* (**D**) regions. For each subplot, the upper part presents the genetic association information of variants on sclerostin within each of the 4 regions. The purple dot is the top association signal in each region. The dots in red to green are those variants in linkage disequilibrium (LD) with the top signal. The middle part presents genes within each of the region. Genes in red are those genes that were mapped to the genetic association signals within this region. Genes in blue are those protein‐coding genes that were not mapped to any of the genetic association signals. Genes in black are those nonprotein coding genes that were not mapped to any genetic signals. The bottom part shows the intersection between genetic association signals and regulatory elements color coded as shown (see Supplementary Table 13 for more details, available on the *Arthritis & Rheumatology* website at http://onlinelibrary.wiley.com/doi/10.1002/art.42538). In subplot **A**, rs215233 is the top hit of the current genome‐wide association study (GWAS) meta‐analysis, and rs215226 the top hit for the previous GWAS meta‐analysis of sclerostin, which is in perfect LD. SNPs = single‐nucleotide polymorphisms.

After applying conditional analysis using GCTA‐COJO, 18 conditionally independent variants within 15 genomic loci were associated with circulating sclerostin (Table 1). The strongest signal, rs215223, was close to the *B4GALNT3* gene (for A allelle, β ± SE −0.136 ± 0.008, *P* = 2.44 × 10^−73^, effect allele frequency = 0.405, variance explained by the variant = 0.89%) (Figure 2A). One *cis*‐acting variant in the *SOST* region, rs66838809, showed a strong association with sclerostin (for A allelle, β ± SE −0.088 ± 0.015, *P* = 1.45 × 10^−9^, effect allele frequency = 0.079, variance explained by the variant = 0.11%; Figure 2B). Another variant, rs28929474 in the *SERPINA1* gene region, was associated with circulating sclerostin (for T allelle, β ± SE 0.173 ± 0.027, *P* = 1.1 × 10^−10^, effect allele frequency = 0.021, variance explained by the variant = 0.12%; Figure 2C). This missense variant constitutes the *PiZ* allele, causing α_1_‐antitrypsin (AAT) deficiency in homozygous cases (42). The variant rs7143806 in the *RIN3* gene region was also associated with sclerostin (β of A allele = 0.053, SE 0.010, *P* = 3.35 × 10^−8^, effect allele frequency = 0.181, variance explained by the variant = 0.08%; Figure 2D). The gene was reported to be associated with lower limb BMD (43). These and the other 14 variants within 12 genomic loci are listed in Table 1. Results of the random effects meta‐analysis were similar to those of the fixed‐effect meta‐analysis (Supplementary Table 5A, http://onlinelibrary.wiley.com/doi/10.1002/art.42538). The degree of heterogeneity was low across studies for most of the identified genetic variants (Table 1). However, we observed evidence for heterogeneity for the *B4GLANT3* variant, rs215223. We found that this variant showed a robust negative effect on sclerostin in all cohorts; however, the genetic effect estimate was particularly large in the 4D cohort, comprising individuals with end‐stage chronic kidney disease (CKD) (Supplementary Table 5B). A possible explanation for this finding is that sclerostin levels are known to be elevated in patients with CKD, presumably reflecting a contribution of renal clearance to circulating levels (44). Nonetheless, this source of variation is unlikely to limit the validity of using *B4GALNT3* to instrument sclerostin levels in the general population, from which other participating cohorts were recruited. As a sensitivity analysis, genetic liability of CKD showed marginal evidence of causal effect on circulating sclerostin (CKD data from CKDGen consortium [45]; Supplementary Table 5C).

**Table 1:** Meta‐analysis results for loci that reached genome‐wide significance*

Locus	SNP	EA	OA	EAF	GENE	*Cis*/*trans*	β	SE	*P*	Q	Q_*P*	I^2^	R^2^
chr1 50566286	rs61781020	A	G	0.049	*FAF1*	*Trans*	0.101	0.018	2.57 × 10^−8^	17.825	0.058	0.439	0.10%
chr2 229236796	rs4973180	T	C	0.821	*PID1*	*Trans*	0.059	0.010	1.04 × 10^−9^	6.691	0.754	0	0.10%
chr5 56813327	rs11960484	A	G	0.351	*MAP3K1*	*Trans*	–0.049	0.008	1.40 × 10^−10^	14.823	0.139	0.325	0.11%
chr5 115994797	rs34498262	A	G	0.391	*LVRN*	*Trans*	0.065	0.008	1.41 × 10^−17^	10.403	0.406	0.039	0.20%
chr5 116013119	rs17138656	A	G	0.124	*LVRN*	*Trans*	0.096	0.011	3.16 × 10^−17^	14.273	0.161	0.299	0.20%
chr6 45189983	rs75523462	T	G	0.950	*SUPT3H*	*Trans*	0.104	0.017	1.31 × 10^−9^	7.424	0.685	0	0.10%
chr6 133044782	rs34366581	T	G	0.326	*LINC00326*	*Trans*	0.047	0.008	2.80 × 10^−9^	13.204	0.212	0.243	0.10%
chr8 119000461	rs11995824	C	G	0.454	*TNFRSF11B*	*Trans*	0.100	0.007	5.62 × 10^−41^	16.222	0.093	0.384	0.49%
chr10 122342063	rs6585816	T	G	0.209	–	*Trans*	0.056	0.009	7.84 × 10^−10^	6.121	0.805	0	0.10%
chr12 481093	rs215223	A	G	0.405	*B4GALNT3*	*Trans*	–0.136	0.008	2.44 × 10^−73^	83.297	1.13 × 10^−13^	0.880	0.89%
chr13 42378009	rs9594738	T	C	0.482	*TNFSF11*	*Trans*	–0.056	0.007	6.48 × 10^−14^	9.217	0.512	0	0.15%
chr13 42513606	rs34136735	T	C	0.052	*TNFSF11*	*Trans*	0.171	0.017	5.69 × 10^−23^	17.750	0.059	0.437	0.29%
chr13 42532378	rs665632	T	C	0.813	*TNFSF11*	*Trans*	0.082	0.010	4.82 × 10^−17^	8.502	0.484	0	0.20%
chr14 92637384	rs7143806	A	G	0.181	*RIN3*	*Trans*	0.053	0.010	3.35 × 10^−8^	13.360	0.204	0.251	0.08%
chr14 94378610	rs28929474	T	C	0.021	*SERPINA1*	*Trans*	0.173	0.027	1.10 × 10^−10^	5.342	0.867	0	0.12%
chr17 43721253	rs66838809	A	G	0.079	*SOST*	*Cis*	–0.088	0.015	1.45 × 10^−9^	13.369	0.147	0.327	0.11%
chr18 62390996	rs2957124	A	G	0.421	*TNFRSF11A*	*Trans*	–0.057	0.008	5.97 × 10^−14^	11.286	0.257	0.203	0.16%
chr20 11231094	rs13042961	T	C	0.955	*JAG1*	*Trans*	0.126	0.019	4.65 × 10^−11^	16.398	0.037	0.512	0.14%

* Genome‐wide significance was defined by *P* < 5 × 10^−8^. Locus refers to the chromosome and position of the SNP; GENE refers to the nearest gene to the sclerostin‐associated SNP. *Cis*/*trans* indicates that the associated SNP is close to the SOST region (noted as *cis*) or far away from this region (noted as *trans*). β indicates the SD change in serum sclerostin per effect allele. Heterogeneity testing was conducted using Cochran's Q statistics (Q) and Cochran's Q *P* value (Q_*P*). R^2^ is the variance explained by each of the top sclerostin variants. SNP = single‐nucleotide polymorphism; EA = effect allele; OA = other allele; EAF = effect allele frequency.

### Genetic colocalization analysis of sclerostin association signals with gene expression

For the 18 sclerostin associated variants, we identified 4 variants (rs215223 in the *B4GALNT3* region, rs28929474 in the *SERPINA1* region, rs66838809 in the *SOST* region, and rs7143806 in the *RIN3* region) where sclerostin‐increasing alleles were associated with lower eBMD (at Bonferroni *P* value cutoff of 0.003) (Supplementary Table 1C and Supplementary Figure 3, http://onlinelibrary.wiley.com/doi/10.1002/art.42538). The genetic colocalization analysis for these 4 variants suggested that the expression of *B4GALNT3* and *SOST* genes showed strong evidence of colocalization with circulating sclerostin levels (colocalization probability 99% and 98%, respectively; Supplementary Table 6A, http://onlinelibrary.wiley.com/doi/10.1002/art.42538). The *SERPINA1* and *RIN3* signal showed weaker evidence of colocalization with sclerostin (Supplementary Figure 4 and Supplementary Table 6A, http://onlinelibrary.wiley.com/doi/10.1002/art.42538). We also confirmed that the *SOST* SNP was associated with altered *SOST* expression in iliac crest bone tissue (Supplementary Table 6B). More details of the other bioinformatics functional follow‐up can be found in Supplementary Note 2 and Supplementary Tables 5A and 7 (http://onlinelibrary.wiley.com/doi/10.1002/art.42538).

### Genetic correlation between sclerostin levels and atherosclerosis‐related traits

As expected, genetic correlation analysis between circulating sclerostin using genetic variants across the whole genome revealed a relationship between lower sclerostin and higher eBMD and, to a lesser extent, lower fracture risk (Supplementary Table 8, http://onlinelibrary.wiley.com/doi/10.1002/art.42538). These analyses also showed a genetic overlap of lower sclerostin with increased hypertension risk (r_g_ = 0.134, *P* = 3.10 × 10^−3^; Table 2), but not with any other atherosclerosis‐related diseases or risk factors (Supplementary Figure 5, http://onlinelibrary.wiley.com/doi/10.1002/art.42538).

**Table 2:** Mendelian randomization and genetic correlation analysis results of the effect of lower sclerostin levels on atherosclerosis and related risk factors*

Exposure	Outcome	Model	N SNPs	OR	LCI	UCI	*P*
Lower sclerostin levels	Type 2 diabetes	*Cis*‐only MR	5	1.32	1.03	1.69	0.030
Lower sclerostin levels	Myocardial infarction	*Cis*‐only MR	5	1.35	1.01	1.79	0.040
Lower sclerostin levels	Coronary artery disease	*Cis*‐only MR	5	1.01	0.74	1.39	0.954
Lower sclerostin levels	Hypertension	*Cis*‐only MR	5	1.02	0.98	1.06	0.311
Lower sclerostin levels	Ischemic stroke	*Cis*‐only MR	2	0.95	0.48	1.87	0.874
Lower sclerostin levels	Cardioembolic stroke	*Cis*‐only MR	2	0.88	0.23	3.35	0.849
Lower sclerostin levels	Large vessel disease	*Cis*‐only MR	2	0.88	0.21	3.75	0.866
Lower sclerostin levels	Small vessel disease	*Cis*‐only MR	2	0.57	0.13	2.60	0.470

Exposure	Outcome	Model	N SNPs	β	SE	*P*
Lower sclerostin levels	Coronary artery calcification	*Cis*‐only MR	5	0.236	0.110	0.033
Lower sclerostin levels	Aortic calcification	*Cis*‐only MR	5	–0.051	0.273	0.851
Lower sclerostin levels	Low density lipoprotein	*Cis*‐only MR	5	–0.008	0.044	0.857
Lower sclerostin levels	High density lipoprotein	*Cis*‐only MR	5	–0.092	0.052	0.073
Lower sclerostin levels	Triglyceride	*Cis*‐only MR	5	0.107	0.082	0.190
Lower sclerostin levels	Apolipoprotein A‐I	*Cis*‐only MR	5	–0.051	0.043	0.236
Lower sclerostin levels	Apolipoprotein B	*Cis*‐only MR	5	0.047	0.044	0.285

Trait 1	Trait 2	Model	N SNPs	r_g_	SE_r_g_	*P*_r_g_
Lower sclerostin levels	Coronary artery calcification	Genetic correlation	All SNPs	0.007	0.083	0.933
Lower sclerostin levels	Hypertension	Genetic correlation	All SNPs	0.134	0.045	3.10 × 10^−3^
Lower sclerostin levels	Type 2 diabetes	Genetic correlation	All SNPs	–0.041	0.073	0.573

Model refers to which statistical method/model was applied. For *cis*‐only analysis, the inverse variance‐weighted Mendelian randomization (MR) method was used. N_SNPs means the number of genetic variants included as predictors for sclerostin. Estimate SE and *P* (and r_g_, SE_r_g_, *P*_r_g_) are the association estimates, SE, and *P* value of the MR (or the genetic correlation analysis). The odds ratio (OR) and 95% confidence interval of the MR estimates using *cis*‐acting variants, which refers to OR of disease risk per SD unit lowering of sclerostin levels, and is not applicable for the genetic correlation analysis. Importantly, the MR and genetic correlation analyses have different assumptions; therefore, the effect estimate is not directly comparable. We listed them in the same table to compare the direction of effects and the *P* value estimates across the 2 approaches. SNP = single‐nucleotide polymorphism; LCI = lower 95% confidence interval; UCI = upper 95% confidence interval.

### Selection of genetic instruments for circulating sclerostin

We considered the *SOST cis* variant and *B4GALNT3*, *SERPINA1*, and *RIN3 trans* variants identified above as possible instruments for MR analyses of the effect of lower sclerostin levels on atherosclerosis risk. The remaining 14 variants identified in our GWAS did not fit with our selection criteria and were therefore excluded from further analysis (Supplementary Figure 3, http://onlinelibrary.wiley.com/doi/10.1002/art.42538). For all 4 SNPs, as expected, the alleles associated with lower circulating sclerostin levels were associated with increased eBMD and reduced fracture risk **(**Supplementary Figure 6, http://onlinelibrary.wiley.com/doi/10.1002/art.42538) and provided strong instrument strength (overall F statistic for the 4 variants = 89.8).

For the *cis*‐only analysis, we included 5 correlated variants (rs66838809, rs1107747, rs4793023, rs80107551, rs76449013; r^2^ < 0.8) that showed associations with circulating sclerostin (*P* < 1 × 10^−6^), where the alleles of these variants associated with lower circulating sclerostin levels were also associated with increased eBMD and reduced fracture risk, which together had acceptable instrument strength (conditional F statistic = 27.7) (Supplementary Table 2, http://onlinelibrary.wiley.com/doi/10.1002/art.42538). Steiger filtering was applied to estimate the directionality of the instruments (46).

To examine possible pleiotropic effects, a phenome‐wide association analysis of these 4 variants was performed, which suggested that the *B4GLANT3*, *RIN3*, and *SOST* variants were additionally associated with lean body mass. We conducted a bidirectional MR of circulating sclerostin and body mass index and found little evidence to support any effect between the two. Therefore, body mass–related traits, including lean body mass, are not likely to be a pleiotropic pathway between sclerostin and atherosclerosis outcomes (Supplementary Table 9A, http://onlinelibrary.wiley.com/doi/10.1002/art.42538). The *RIN3* variant was also related to hemoglobin A1c, endometriosis, and breast cancer. The *SERPINA1* variant was relatively pleiotropic, being associated with a range of traits including sex hormone–binding globulin levels, total testosterone, cholelithiasis, chronic obstructive pulmonary disease, CAD, and prostate cancer (Supplementary Table 9B). We also examined potential pleiotropy by conducting a proteome‐wide association scan of the 4 genetic variants. Sclerostin variants within *SOST* were not associated with any other proteins. In contrast, the *RIN3* region was associated with 1 other protein, and variants within *B4GLANT3* and *SERPINA1* regions were associated with an additional 16 and 58 proteins, respectively (Supplementary Table 9C).

### Effects of lower sclerostin on risk of atherosclerosis‐related diseases and risk factors

We used the 5 correlated *cis*‐acting *SOST* instruments to evaluate causal effects of lower sclerostin levels on 15 atherosclerosis‐related diseases and risk factors. The IVW analysis identified potential adverse effects of lower sclerostin on increased risk of type 2 DM (OR 1.32 [95% CI 1.03–1.69]) and MI (OR 1.35 [95% CI 1.01–1.79]; Table 2 and Supplementary Figure 7, http://onlinelibrary.wiley.com/doi/10.1002/art.42538). Genetically predicted lower sclerostin showed an effect on increasing levels of CAC (β = 0.24 [95% CI 0.02–0.45]) (Table 2). Results of the MR Egger analyses are shown in Supplementary Table 10 (http://onlinelibrary.wiley.com/doi/10.1002/art.42538). Heterogeneity analysis of MR estimates of each genetic instrument suggested little evidence of heterogeneity across the 5 genetic instruments (Cochran's Q test *P* > 0.05; Supplementary Table 10, http://onlinelibrary.wiley.com/doi/10.1002/art.42538). In contrast, we observed little evidence of a causal effect of lower sclerostin on AAC, CAD, risk of stroke (and its subtypes), risk of hypertension, and lipid subtypes.

We also examined causal effects of sclerostin using a *cis+trans* genetic instrument which also included *B4GALNT3*, *SERPINA1*, and *RIN3* SNPs. The MR effects on stroke were estimated using 2 variants in the *B4GLANT3* and *RIN3* regions, where the genetic association information of the other 2 variants were missed in the stroke outcome datasets. Lower circulating sclerostin was associated with an increased risk of hypertension (OR 1.09 per SD decrease in sclerostin [95% CI 1.04–1.15]; *P* = 7.93 × 10^−4^), whereas the effects are generally attenuated for other outcomes in the *cis*+*trans* analyses (Supplementary Figures 7 and 8, http://onlinelibrary.wiley.com/doi/10.1002/art.42538). Sensitivity analyses suggested little evidence of horizontal pleiotropy (Egger regression intercept = –0.003, *P* = 0.27) or heterogeneity (Cochran's Q = 2.85, *P* = 0.42; Supplementary Table 9D, http://onlinelibrary.wiley.com/doi/10.1002/art.42538). In contrast, little evidence for a causal effect of lower sclerostin on any other atherosclerosis‐related disease or risk factor was identified (Supplementary Table 9D and E).

### Effects of atherosclerosis‐related diseases and risk factors on circulating sclerostin

We further conducted bidirectional MR to evaluate the potential reverse causality of 15 atherosclerosis‐related diseases and risk factors on circulating sclerostin (instruments listed in Supplementary Table 4, http://onlinelibrary.wiley.com/doi/10.1002/art.42538). A marginally positive relationship for liability to type 2 DM on sclerostin was observed (β = 0.02, SD change in sclerostin per unit increase of risk score of type 2 DM [95% CI 0.001–0.045]; *P* = 0.04, Supplementary Table 11A, http://onlinelibrary.wiley.com/doi/10.1002/art.42538). Apo B showed a negative effect on sclerostin levels (β = –0.03 [95% CI –0.01, –0.06]; *P* = 3.67 × 10^−3^). However, the multivariable MR including Apo B, LDL cholesterol, and triglycerides in the same model suggested that increased Apo B levels increased sclerostin levels (β = 0.03 [95% CI 0.001–0.07]; *P* = 0.041, Supplementary Figure 8 and Supplementary Table 11B, http://onlinelibrary.wiley.com/doi/10.1002/art.42538). Genetic liability to atherosclerosis‐related diseases or risk factors showed little evidence of a reverse effect on sclerostin (Supplementary Table 11A). As a validation, we estimated the effect of eBMD and liability to fracture on circulating sclerostin, observing a strong positive effect of eBMD on sclerostin (Supplementary Table 11A), consistent with findings from our previous sclerostin study (10). Sensitivity analyses provided little evidence to suggest directional pleiotropy or heterogeneity of the causal estimates (Supplementary Table 11A). The Steiger filtering analysis further confirmed that the sclerostin instruments were likely to first change the sclerostin level and then influence the atherosclerosis outcomes as a causal consequence (Supplementary Table 11C).

## DISCUSSION

We have presented findings from an updated GWAS meta‐analysis of circulating sclerostin, which identified 18 sclerostin‐associated variants, of which 4 in the *SOST*, *B4GALNT3*, *RIN3*, and *SERPINA1* genes provided useful genetic instruments for determining the causal effects of lower sclerostin levels on atherosclerosis‐related diseases and risk factors based on inverse relationships between sclerostin levels and BMD. Lower sclerostin levels showed a causal effect on hypertension risk using the combined *cis* and *trans* instruments, without evidence of reverse causality. We found that *cis*‐only analyses suggested causal effects of lower sclerostin levels on atherosclerosis‐related diseases and type 2 DM, and in particular that lower levels of sclerostin increases risk of MI and increases the extent of CAC. However, whereas the *cis* instrument suggested a causal effect of sclerostin lowering on CAC and MI, there was no equivalent effect on AAC or stroke.

These findings are in part consistent with those of 2 previous phase III trials with the sclerostin inhibitor romosozumab, which found an increased event rate for MI in those randomized to active treatment in postmenopausal women (1) and in men (2). That said, these trials also found an increased signal for stroke, whereas MR analyses in the present study were close to the null (although estimated with little precision) with respect to stroke. Thus, one potential explanation for this apparent discrepancy is that our analyses for stroke had limited power, with both *cis*‐only and *cis+trans* analyses based on only 2 SNPs as the remaining SNPs were missing in the outcome GWAS dataset. Alternatively, suggestions of increased stroke risk in these 2 trials may have been spurious due to chance fluctuations in low absolute event rates, and equivalent findings were not observed in a third phase III trial (1).

*Cis* instruments are more likely to directly link with biology, which aligns with our finding that *cis*‐only analyses identified effects of lower sclerostin on MI risk, extent of CAC, and risk of type 2 DM, whereas these were not seen in our *cis+trans* analyses. On the other hand, our finding that sclerostin lowering only increased the risk of hypertension when using the *cis+trans* instrument could result from pleiotropy. *Trans* instruments are, by their nature, more likely to be pleiotropic, which was supported by findings from phenome‐ and proteome‐wide analyses suggesting that all 3 *trans* instruments selected had a high potential for pleiotropy. Additionally, *cis* variants may be better predictors of sclerostin levels in tissues responsible for mediating biological effects. Based on eQTL data using bone tissue, the *cis* signal is predicted to alter expression and hence local levels of sclerostin in bone cells. Osteocytes, embedded within bone and constituting approximately 80% of bone cells, are the primary source of sclerostin, which then circulates locally through canaliculi to modulate the activity of other bone cells, including osteoblasts, leading to changes in bone mass and strength (47). Accordingly, the *cis* signal is expected to alter circulating levels of sclerostin through exchange between bone tissue and the circulation. In contrast, we previously hypothesized that the *trans* signal *B4GALNT3*, replicated in the present study, primarily influences circulating sclerostin levels by affecting plasma clearance due to altered protein glycosylation (10). Hence, any changes in tissue sclerostin levels resulting from the *B4GALNT3 trans* signal are likely to be secondary to altered circulating levels, rather than local production. Therefore, by its nature, the B4GALNT3 *trans* signal is expected to produce smaller changes in tissue sclerostin levels compared to a *cis SOST* signal, leading to a weaker effect on eBMD.

That the *SOST cis* signal is likely to produce greater increases in tissue sclerostin levels compared to *trans* signals, provides an explanation as to why the *cis*‐only analyses predicted more extraskeletal effects of sclerostin lowering compared to the *cis+trans* analyses. Sclerostin is also expressed in vascular tissues including at sites of vascular calcification (48), suggesting that any effects of sclerostin on vascular tissues may also involve local sclerostin expression. Such an effect is likely mediated by sclerostin's well‐recognized action as a WNT inhibitor (49), given the contribution of WNT signalling to the development of atherosclerosis (50).

Pharmacokinetic studies suggest that romosozumab is largely retained within the circulation (51), in keeping with the relatively large size of a monoclonal antibody. That said, the pharmacologic action of romosozumab (involving neutralization of sclerostin activity in bone tissue) depends on the antibody‐penetrating skeletal tissue after systemic administration, which is likely to involve convection or endocytosis/pinocytosis via endothelial cells (52). To the extent that effects of romosozumab on CVD risk also involve local tissue penetration, a *cis* instrument reflecting tissue levels of sclerostin may be more likely to predict effects of romosozumab on CVD risk than a *trans* instrument more closely linked to systemic levels.

There have also been several previous observational studies examining associations between circulating sclerostin and atherosclerosis‐related diseases and risk factors. Our recent observational study found observational associations in the opposite direction to those causal effects predicted by our MR analyses (53), particularly in analyses restricted to the *cis* instrument. Interestingly, directionally opposite effects have also been observed in the case of eBMD and atherosclerosis risk, with a protective effect found in an observational analysis but a harmful effect predicted by MR analyses (53). The latter finding also raises the possibility that any effect of sclerostin lowering on atherosclerosis risk might be an indirect consequence of increased BMD, as opposed to a specific effect of sclerostin. However, arguing against this suggestion, there is little evidence that other therapeutic agents for osteoporosis acting to increase BMD affect atherosclerosis risk, apart from strontium ranelate for which the European Medicines Agency issued a warning, restricting use in those with a high risk of CVD (54).

Two previous studies have used MR approaches to examine causal effects of sclerostin lowering on atherosclerosis and related risk factors. Bovijn et al reported that 2 conditionally independent *SOST* SNPs, selected on the basis of their association with eBMD, predicted higher risk of MI and/or coronary revascularization, major cardiovascular events, hypertension, and type 2 DM (8). Our MR finding on MI, using the *cis*‐only instrument for circulating sclerostin, is consistent with these observations. In contrast, Holdsworth et al found no association between gene expression level of *SOST* in tibial artery/heart tissue and risk of CVD, using 3 *cis SOST* eQTLs as instruments (9). Despite the distinct methods used to proxy sclerostin lowering, our *cis* instrument is in strong LD with those used in these other studies. Indeed, our *cis* instrument shares an identical SNP with the Holdsworth study (see Supplementary Table 12, http://onlinelibrary.wiley.com/doi/10.1002/art.42538). In terms of explanations for the differences observed, eQTL data from Holdsworth et al were based on tibial artery/heart tissues, whereas circulating sclerostin as measured in the present study is mainly derived from bone, so different findings likely reflect distinct genetic regulatory mechanisms between different tissues. Given the known relationship between bone and glucose metabolism (55), the potential adverse effect of lower levels of sclerostin on type 2 DM also need further investigation in future randomized clinical trials.

In terms of other *trans*‐acting pathways, we have identified 2 new *trans* signals for sclerostin, *RIN3* and *SERPINA1*. Previous GWASs have identified *RIN3* in association with lower limb and total BMD in children (43), and Paget's disease of bone (56). Homozygosity of *SERPINA1* underlies deficiency of AAT, a glycoprotein mostly produced by the liver, which serves to protect lung tissue from tissue damage caused by proteases released from neutrophils. The loss of function allele was associated with higher sclerostin levels, and the mechanisms underlying this genetic association are unclear. AAT deficiency causes early‐onset COPD (57); however, we are not aware of any previous findings relating AAT to BMD or risk of osteoporosis. Given the lack of evidence of colocalization, it is also possible that a different gene was responsible for the genetic signal identified at this locus.

In terms of strengths, the present study had sufficient sample size to clearly detect a *cis (SOST)* signal, and our genetic instrument successfully accounted for bidirectional effects between sclerostin and BMD, by removing *trans* SNPs with the same direction of effect on sclerostin and eBMD. Our MR of sclerostin effects on atherosclerosis‐related diseases and risk factors used circulating level of sclerostin as the exposure, which may predict adverse effects from sclerostin antibody inhibition more accurately than previous studies using BMD or *SOST* arterial expression as exposures. Finally, since genetic predictors in the *cis*‐ and/or *trans*‐acting regions may yield different causal estimates on outcomes, we considered these separately. In terms of weaknesses, though postmenopausal women are the main target group for osteoporosis treatments such as romososumab, we were only able to examine predicted effects of sclerostin lowering in males and females combined, due to the lack of availability of sex‐specific sclerostin GWAS dataset. In addition, the different cohorts used distinct methods to measure sclerostin, with the over half providing sclerostin measures through the SomaLogic platform, while the other half used a specific ELISA. However, despite these methodologic differences, there was little evidence of heterogeneity of genetic associations between cohorts. A further limitation is that we did not apply Bonferroni correction to account for testing multiple outcomes in our MR analyses, inclusion of which would have raised the *P* values attached to the findings from *cis*‐only analyses. That said, though there was only moderate evidence supporting our observations for MI risk, this was one of the key outcomes of our study given findings from previous clinical trials, and findings should be considered within a triangulation of evidence framework (58).

In conclusion, our updated GWAS meta‐analysis of circulating sclerostin now identified a robust *cis* (*SOST*) signal, replicated our previous *B4GALNT3* signal, and identified new *trans* signals in the *RIN3* and *SERPINA1* genes. Genetically predicted lower sclerostin levels were found to associate with higher risk of hypertension, MI, type 2 DM, and increased CAC. To the extent that genetically predicted lower lifelong exposure to sclerostin shares consequences with pharmacologic inhibition over 12 months, our results underscore the requirement for strategies to mitigate potential adverse effects of romosozumab treatment on atherosclerosis and its related risk factors.

## AUTHOR CONTRIBUTIONS

All authors were involved in drafting the article or revising it critically for important intellectual content, and all authors approved the final version to be published. Dr. Zheng had full access to all of the data in the study and takes responsibility for the integrity of the data and the accuracy of the data analysis.

### Study conception and design

Zheng, Davey Smith, Tobias.

### Acquisition of data

Zheng, Wheeler, Pietzner, Andlauer, Yau, Brumpton, Rasheed, Maerz, Peyser, Kavousi, Vries, Zeggini, Davey Smith, Tobias.

### Analysis and interpretation of data

Zheng Wheeler, Pietzner, Andlauer, Yau, Hartley, Brumpton, Rasheed, Kemp, Frysz, Robinson, Reppe, Prijatelj, Gautvik, Falk, Maerz, Gergei, Peyser, Kavousi, de Vries, Miller, Bos, van der Laan, Malhotra, Herrmann, Scharnagl, Kleber, Dedoussis, Zeggini, Nethander, Ohlsson, Lorentzon, Wareham, Langenberg, Holmes, Davey Smith, Tobias.

## Supporting information


Disclosure Form

**Appendix S1:** Supplementary File

**Supplementary Table 1A** Selection of genetic predictors for sclerostin inhibtion.
**Supplementary Table 1B**. Selection of genetic predictors for sclerostin inhibtion.
**Supplementary Table 1C**. Genetic predictors of sclerostin after selection.
**Supplementary Table 2**. Cis‐only genetic instruments of circulating sclerostin (Selection citeria: P<1E‐7, r2<0.8)
**Supplementary Table 3**. Details of outcomes relating to atherosclerosis traits and its related risk factors.
**Supplementary Table 4A**. Genetic instruments of atherosclerosis traits and related risk factors.
**Supplementary Table 4B**. Genetic instruments for HDL‐C and apoA‐I used in the multivariable MR model.
**Supplementary Table 4C**. Genetic instruments for LDL‐C, TG and apoB used in the multivariable MR model.
**Supplementary Table 5A**. Genetic variants associated with circulating sclerostin at genome‐wide significance
**Supplementary Table 5B**. Genetic variants associated with circulating sclerostin in each cohort.
**Supplementary Table 5C**. Effect of genetic liability of chronic kidney disease on circulating sclerostin.
**Supplementary Table 6A**. Genetic colocalization of sclerostin on expression of four genes.
**Supplementary Table 7**. Chromatin accessibility analysis predicted by ChromHMM.
**Supplementary Table 8**. LD score regression results of sclerostin (lowering) on 15 atherosclerosis related outcomes and two bone phenotypes.
**Supplementary Table 9A**. Bidirectional Mendelian randomization of BMI against circulating sclerostin.
**Supplementary Table 9B**. Phenome‐wide association results of the four sclerostin instruments.
**Supplementary Table 9C**. Association of sclerostin variants with other plasma protein levels.
**Supplementary Table 9D**. Mendelian randomization estimates of sclerostin (lowering) on atherosclerosis traits and related risk factors using both cis‐ and trans‐acting genetic instruments
**Supplementary Table 9E**. Mendelian randomization estimates of sclerostin (lowering) on atherosclerosis traits and related risk factors using both cis‐ and trans‐acting genetic instruments excluding varaints in the *B4GLANT3* or *SERPINA1* region.
**Supplementary Table 10**. Mendelian randomizaiton estimates of sclerostin (lowering) on atherosclerosis traits and related risk factors using only cis‐acting genetic instruments
**Supplementary Table 11A**. Reverse Mendelian randomization estimates of atherosclerosis traits/related risk factors on circulating sclerostin.
**Supplementary Table 11B**. Multivariable Mendelian randomization of lipids phenotypes on circulating sclerostin.
**Supplementary Table 11C**. Steiger filtering estimates of sclerostin instruments on atherosclerosis traits and related risk factors.
**Supplementary Table 12**. LD between the cis‐acting instruments of sclerostin that have been used in this study and two other studies using cis‐acting sclerostin variants*
**Supplementary Table 13**. Description of the regulation elements included in the region plots.
